# Comprehensive comparison of clinicoradiological, laboratory, and prognostic factors of community-acquired pneumonia in diabetic and nondiabetic hospitalized patients

**DOI:** 10.55730/1300-0144.5747

**Published:** 2023-10-10

**Authors:** Seyyed Hamid HASHEMI, Fatemeh SAKI, Shiva BORZOUEI, Rashed BAWAND, Alireza SOLTANIAN

**Affiliations:** 1 Department of Infectious Diseases, School of Medicine, Hamadan University of Medical Sciences, Hamadan, Iran; 2 Department of Infectious Diseases, School of Medicine, Hamadan University of Medical Sciences, Hamadan, Iran; 3 Department of Endocrinology, School of Medicine, Hamadan University of Medical Sciences, Hamadan, Iran; 4 Department of General Medicine, School of Medicine, Hamadan University of Medical Sciences, Hamadan, Iran; 5 Department of Biostatics, School of Public Health, Hamadan University of Medical Sciences, Hamadan, Iran

**Keywords:** Pneumonia, respiratory, infection, diabetes mellitus, clinical, prognosis

## Abstract

**Background/aim:**

Community-acquired pneumonia (CAP) is one of the leading infectious causes of mortality, and diabetes mellitus is a globally prevalent disease. Consequently, the cooccurrence of these two disorders can be common and create challenging medical conditions. Therefore, it was aimed to compare the various aspects of CAP in diabetic and nondiabetic patients, in order to have a comprehensive and comparative picture of the differences.

**Materials and methods:**

In this cross-sectional study, CAP patients with and without diabetes were assessed for clinicoradiological signs, laboratory features, disease severity, and pneumonia outcomes.

**Results:**

Analyzed herein were 172 CAP patients (77 had diabetes and 95 were nondiabetic). Clinical and radiological signs of pneumonia were mostly similar between the groups, except for purulent sputum, which was more prevalent among the nondiabetic patients. The laboratory results were also mostly similar. However, analysis of the outcomes and prognosis showed different results. The diabetic patients had a longer mean duration of hospital stay (8.52 days vs. 7.93 days, p = 0.015), higher median pneumonia severity based on the CURB-65 criteria (3 vs. 2, p = 0.016), and higher intensive care unit (ICU) admission requirement (22.1% vs. 7.3%, p = 0.004). Moreover, the mortality rate for the diabetic patients was nonsignificantly higher (16.8% vs. 15.7%, p = 0.453). Furthermore, the results of the logistic regression analysis showed that the diabetic patients had significantly higher odds of experiencing more severe forms of pneumonia (adjusted odds ratio (AOR): 5.77, 95% CI: 2.52–13.20), requiring ICU hospitalization (AOR: 3.56, 95% CI: 1.39–9.11), and having a longer hospital stay (AOR: 2.01, 95% CI: 1.09–3.71). In addition, although there was no significant relationship between the severity of pneumonia and the amount of glycated hemoglobin (HbA1c) in the diabetic patients (p = 0.940), the higher level of HbA1c in the nondiabetic patients was significantly correlated with a higher severity of pneumonia (p = 0.002).

**Conclusion:**

While diabetic patients with CAP have the same clinicoradiological and laboratory features as nondiabetic patients, the presence of diabetes can significantly worsen the outcomes and prognosis of pneumonia.

## 1. Introduction

Pneumonia is a prevalent infection and ranks among the leading causes of death globally, particularly in the elderly population [1]. According to previous studies, pneumonia can be considered the leading cause of death for pulmonary reasons after lung cancer in some areas [2]. In ambulatory settings, the mortality rate for community-acquired pneumonia (CAP) is below 3%; however, for patients admitted to general wards, this rate escalates to 5%–10% and can surge to as high as 25% for those in the intensive care unit (ICU) [3]. It is important to note that while fatalities associated with lower respiratory tract infections are more widespread in low-income nations, pneumonia stands as the sole infection among the top ten leading causes of death in high-income countries [4]. For example, in the USA, pneumonia is the leading mortality-related infection and the sixth overall reason for death [5]. In Iran, it is estimated that pneumonia is responsible for 9733 deaths annually across all age groups, representing the largest proportion of mortality due to infection [6].

On the other hand, the global prevalence of diabetes has increased dramatically, from approximately 30 million in 1985 to 415 million in 2017 [1], and diabetes is one of the most important causes of immunosuppression [7]. Therefore, diabetic patients are more prone to various types of infections [8–11]. Moreover, in the case of infection, the severity of the disease and the degree of mortality in diabetic patients can be much higher than in the general population [8,12,13]. For these reasons, the accompaniment of pneumonia with diabetes mellitus is both common and challenging.

Although the organisms that cause pneumonia in diabetic patients are mostly similar to those that cause pneumonia in the normal population (except for the increased prevalence of gram-negative bacillus, staphylococcus aureus, and mycobacterium tuberculosis in diabetic patients) [1], some studies have indicated that diabetic patients may experience increased severity, complications, and mortality from pneumonia [14,15].

Therefore, this study was conducted to compare the clinical, paraclinical, and prognostic features of pneumonia in diabetic and nondiabetic patients to have a better understanding of these differences and provide better health care. Previous studies that have been conducted in this field have often focused on the comparison of only one aspect of CAP between diabetic and nondiabetic patients, but in this study, it was attempted to compare all of the clinical, paraclinical, and prognostic dimensions of CAP between diabetic and nondiabetic patients in order to obtain a comprehensive picture of CAP differences between these groups.

## 2. Materials and methods

### 2.1 General principles

This research was a descriptive cross-sectional study with accessible sequential census sampling that included 172 eligible patients. Ethics committee and Institutional Review Board approval for the study were obtained from Hamadan University of Medical Sciences (No.: 980210736). Informed consent was obtained from all of the participants and the ethical principles of the Declaration of Helsinki were adhered to throughout the study. Nonparticipation in the study did not affect the diagnostic or therapeutic processes of the patients. Data were collected without including names and personal details, and the results were not provided to any specific person or organization.

### 2.2. Inclusion and exclusion criteria

All patients aged 18 years and older who were admitted with a definite diagnosis of CAP [1] at the Infectious Ward of Sina Hospital of Hamadan, Iran, from April 2020 to July 2021, were included. Excluding criteria were a diagnosis of pulmonary thromboembolism, myocardial infarction, or pulmonary tuberculosis, and having an uncertain condition of diabetes.

### 2.3. Definitions

- ➢ Pneumonia diagnosis: The diagnosis of CAP was established upon the identification of new lung infiltrates on the chest X-ray (CXR) graph, accompanied by at least one of the following criteria: a fever exceeding 38 °C, hypothermia, alterations in lung auscultation, or respiratory symptoms such as cough, sputum production, dyspnea, or pleuritic discomfort [1].
- ➢ The severity of pneumonia was measured by the CURB-65 criteria [1]. The name of these criteria is an acronym for each one of the mortality and morbidity risk factors of CAP. Each risk factor scores one point, for a maximum score of 5:
  I. Acute confusion
  II. Blood urea nitrogen (BUN) more than 19 mg/dL (7 mmol/L)
  I. Respiratory rate of 30 breaths/min or more
  II. Blood pressure less than 90 mmHg systolic or diastolic blood pressure 60 mmHg or less
  III. Age 65 or older
- ➢ Diabetes status:
  - Diabetic patients: Only patients who had been given a definitive diagnosis of diabetes by a physician, before having pneumonia, and had complete medical evidence of diabetes were considered as diabetic.
  - Nondiabetic patients [1]: Patients who have never been diagnosed with diabetes and have not used any blood sugar-lowering medications, who in the first blood test (before receiving any medication), had a fasting blood glucose (FBG) level <100 mg/dL and glycated hemoglobin (HbA1c) level <5.7%, were considered as nondiabetic.
  - Patients with an uncertain condition of diabetes: Patients with any blood sugar condition other than those mentioned previously were considered to have an uncertain condition of diabetes and were excluded from the study.
- ➢ Recovery status:
  - Complete recovery: Having no pneumonia-related morbidities at the time of discharge from the hospital.
  - Relative recovery: Having any pneumonia-related morbidity at the time of discharge from the hospital.

### 2.4. Procedures

Demographic data of the patients were recorded. Furthermore, the past medical histories and the presence of underlying diseases were evaluated. A CXR graph was taken of all of the patients, and the clinicoradiological signs, as well as the disease course, were recorded.

Before starting the medical treatments, including antibiotics, a blood sample was sent for culture. Another sample was taken to measure the FBG and HbA1c, complete blood count (CBC), C-reactive protein (CRP), erythrocyte sedimentation rate (ESR), and BUN and creatinine levels. Moreover, a sputum (or respiratory secretions suction) sample was sent for smear and culture analysis.

### 2.5. Statistical analysis

After collecting the data, it was entered into IBM SPSS Statistics for Windows 26.0 (IBM Corp., Armonk, NY, USA) and Comprehensive Meta-Analysis 3.0 (Biostat, Englewood, NJ, USA). Central and dispersion indices were then used to express the quantitative variables, while ratios and rates were used to express the qualitative data. This data was then summarized using tables and graphs. To determine the differences between the diabetic and nondiabetic patients in various areas, appropriate statistical tests such as the Kruskal–Wallis, Pearson’s chi-squared (or Fisher’s exact test), and Mann–Whitney U tests were utilized. All of the tests were 2-tailed, and p < 0.05 was considered statistically significant. Moreover, the adjusted odds ratios (AORs) of having diabetes among patients with worse pneumonia outcomes and poorer prognosis were calculated using a logistic regression model.

## 3. Results

### 3.1. General findings

In this study, 172 patients with pneumonia were evaluated, of whom 77 (44.8%) had diabetes, and 95 (55.2%) did not. The mean age of the patients was 66.82 ± 16.91 years, with a minimum of 18 years, and a maximum of 96. Furthermore, the mean ages of the diabetic and nondiabetic patients were 68.74 ± 11.19 and 65.27 ± 20.34 years, respectively (p = 0.182). Of the 172 patients with pneumonia, 79 (45.9%) were male and 93 (54.1%) were female. On the other hand, 41.6% of the males had diabetes and this rate for the females was 49.5% (p = 0.300). Moreover, the frequency of smoking in the nondiabetic patients (p = 0.014) and history of cardiac diseases in the diabetic patients (p = 0.040) were significantly higher (Table 1).

### 3.2. Clinicoradiological evaluations

As shown in Table 1, there was no significant difference between the diabetic and nondiabetic patients with pneumonia in terms of most of the clinical signs and symptoms, except for the frequency of purulent sputum, which was significantly higher in the nondiabetic patients (p = 0.003), which can be attributable to a higher rate of smoking in this group. The radiological signs of pneumonia on the CXRs of both groups were also similar, and the frequency of various lung infiltration patterns (interstitial, lobular, and lobar), atelectasis, bronchopneumonia, and pneumatocele did not differ significantly between them (Table 1).

### 3.3. Laboratory findings

The mean HbA1c rate, and FBG and random blood glucose (RBG) levels were significantly higher in the diabetic patients (Table 1). However, there was no significant difference between these groups in terms of the BUN and creatinine levels or the ESR and white blood cell (WBC) count (Table 1). In addition, as shown in Table 2, there was no significant difference between the diabetic and nondiabetic patients with pneumonia in terms of the sputum smear and culture, blood culture, or blood CRP level.

### 3.4. Prognostic factors

**Figure 1:**
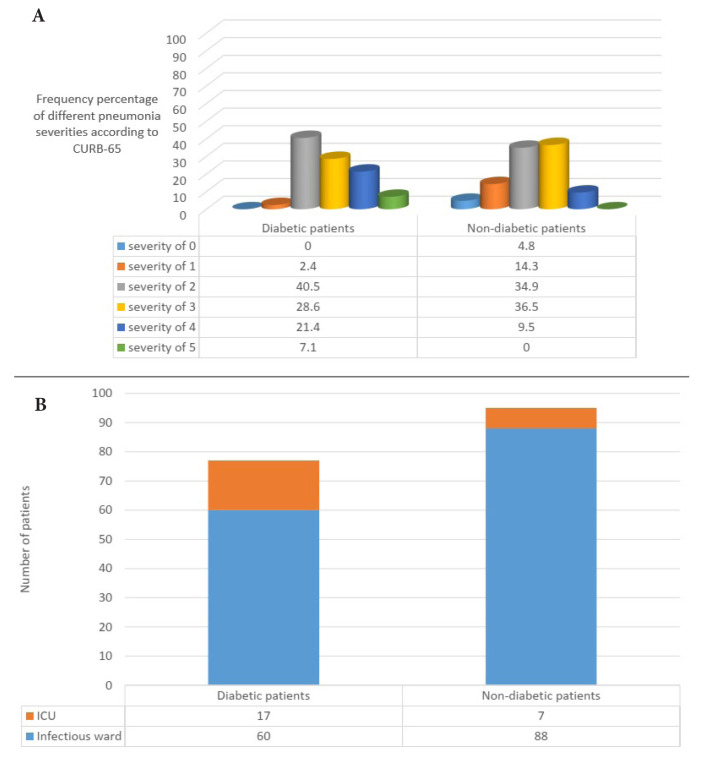
Pneumonia severity indices of the diabetic and nondiabetic patients: A: pneumonia severity according to the CURB-65 criteria (numbers in the table are the frequency percentages), and B: the need for ICU admission.

As shown in Figure 1A, the median pneumonia severity, according to the CURB-65 criteria, in the diabetic and nondiabetic patients was 3 and 2, respectively, and it was significantly higher in the diabetic patients (p = 0.016). Furthermore, 7.1% of the diabetic patients scored 5, but none of the nondiabetic patients had this score. On the other hand, 4.8% of the nondiabetic patients had pneumonia with a severity of 0, while none of the diabetic patients had this score.

Moreover, as presented in Figure 1B, 22.1% of the 77 diabetic patients, and 7.3% of the 95 nondiabetic patients were admitted to the ICU as a result of a clinical decision made by their physicians, and the frequency of diabetic patients requiring ICU hospitalization was significantly higher (p = 0.004).

Additionally, according to the findings in Table 3, there was no significant relationship between the severity of pneumonia and the HbA1c rate (as the long-term indicator of blood sugar control status) in the diabetic patients. Nevertheless, the mean HbA1c level in the nondiabetic patients was correlated with the severity of pneumonia (Table 3). The results of the Tukey post hoc test showed that the HbA1c level was significantly higher in patients with a pneumonia severity of 4 than in those with a severity of 0 (p = 0.002), 1 (p < 0.001), 2 (p = 0.010), and 3 (p = 0.003). These results could mean that higher serum Hb1Ac levels in nondiabetic patients with pneumonia can be considered as a poor prognostic factor. However, the mean HbA1c level in nondiabetic patients with a severity of 0, 1, 2, and 3 did not show a significant difference (p > 0.05).

**Figure 2:**
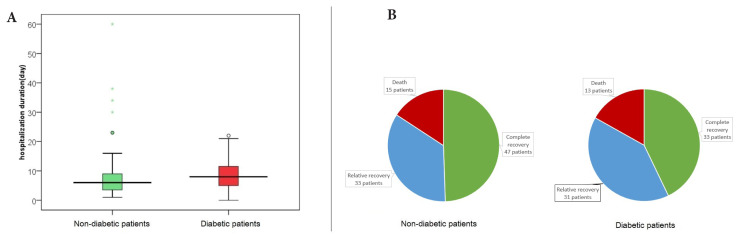
Distribution of the hospitalization duration (A) and pneumonia outcomes (B) in the diabetic and nondiabetic patients.

The mean length of hospital stay for the diabetic patients was 8.52 days, with a median of 8 days, a minimum of 1 days, and a maximum of 22 days (Figure 2A), while for the nondiabetic patients, the mean length of hospital stay was 7.93 days, with a median of 6 days, and minimum 1 and maximum of 60 days (Figure 2A); and the mean admission time of the diabetic patients was significantly higher (p = 0.015).

According to the findings in Figure 2B, among the diabetic patients, 42.85% were discharged with complete recovery, 40.25% with relative recovery, and 16.88% died. These rates for the nondiabetic patients were 49.47%, 34.73%, and 15.78%, respectively, and there was no significant difference between the diabetic and nondiabetic patients with pneumonia in terms of the pneumonia outcomes (p = 0.453).

**Figure 3:**
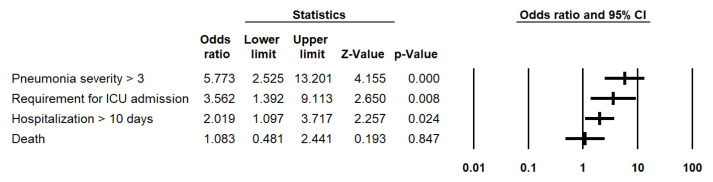
AORs (logistic regression model) for having diabetes among patients with more severe pneumonia (according to the CURB-65 criteria), patients who required ICU admission, patients with a longer hospital stay, and patients who died due to pneumonia.

Moreover, an analysis employing a logistic regression model was conducted to independently evaluate the distinct prognostic factors associated with pneumonia. This approach ensured that the influence of the other variables did not confound the results. Specifically, AORs were calculated to elucidate the correlation between diabetes and various adverse prognostic indicators of pneumonia, including a higher CURB-65 score, ICU admission, extended duration of hospitalization, and mortality in pneumonia-afflicted patients. The outcome of these analyses is showcased in Figure 3, which clearly indicates that diabetic patients have significantly higher odds of experiencing more severe forms of pneumonia, requiring ICU hospitalization, and longer hospital stays compared to the nondiabetic group. However, there was no significant difference observed between the groups in terms of the likelihood of death resulting from pneumonia.

## 4. Discussion

In the present study, 44.8% of the CAP patients had diabetes. The prevalence of diabetes in patients with pneumonia in the study of Rueda et al. [16] was 22.7%, while Chen et al. [17] reported 27.9% and Ibrahem et al. [18] reported 39.8%. These findings indicate the high prevalence of pneumonia in people with diabetes and emphasize the importance of this subject.

Based on the findings of the current study, the age and sex distributions of the diabetic and nondiabetic patients were not significantly different. In some similar studies, the mean age of diabetic patients with pneumonia was higher [14,19]. These discrepancies may be due to differences in the sample sizes and prevalence of diabetes in different communities.

In the present study, in terms of underlying diseases and predisposing factors for pneumonia, the frequency of heart disease was higher in the diabetic patients, while the history of smoking was higher in the nondiabetic group. Similar results have been reported in some other studies [5,19].

In terms of clinical symptoms, the only significant difference was the presence of a more productive cough in the nondiabetic group. Similarly, Fernández et al. [19] declared that medical conditions did not vary significantly between CAP patients with or without diabetes, and Kornum et al. [20] stated that type 2 diabetes was not associated with lung problems or blood infections in patients with pneumonia. However, in the study of Khosh Khoy et al. [5], fever (p < 0.0001) and dyspnea (p = 0.03) were significantly more frequent in diabetic patients, and results reported by Ibrahem et al. [18] showed a higher rate of consciousness impairment in the diabetic group.

There was no significant difference between the groups in terms of radiological manifestations in the current study. Nevertheless, in the studies of Bhambar et al. [21] and Saibal et al. [22], multiple lobular infiltrations were more prevalent in diabetic patients. Furthermore, Khosh Khoy et al. [5] reported a higher incidence of pleural effusion (p = 0.002) and lobar consolidation (p = 0.02) in patients with diabetes.

In terms of pneumonia severity according to the CURB-65 score, in the current study, the diabetic patients had more severe pneumonia than the nondiabetic patients. In the study of Falguera et al. [14], most of the diabetic patients had pneumonia with a severity of 4 and most of the nondiabetic patients had pneumonia with a severity of 1. Additionally, similar results were seen in the study of Ibrahem et al. [18].

A study to determine the association of HbA1c levels with the risk of hospitalization in pneumonia patients showed that poor hyperglycemic control in diabetic individuals increases the risk of hospitalization [23]; moreover, in the study of Ibrahem et al. [18], the CURB-65 score had a significant positive correlation with the HbA1c levels, and Hirata et al. [24] concluded that the serum glucose level at the time of admission (p = 0.01), and the average serum glucose level during hospital stay (p < 0.0001) had significant relationships with the death rate within 30 days for patients with pneumonia, and this relationship remained significant (p = 0.004) even after considering other factors that affect the severity of pneumonia. Furthermore, according to the study of Liu et al. [25], the rate of stress hyperglycemia was related to systematic inflammation and had a J-shaped relationship with negative clinical outcomes in diabetic patients with pneumonia of varying severity. However, contrary to previous studies, the current research found no correlation between the severity of pneumonia and the HbA1c levels in the diabetic patients. Of course, the results herein confirmed that the glycemic status in nondiabetic patients is statistically correlated with pneumonia severity. Therefore, the observed discrepancy in the analysis of the diabetic patients in the present study may have been due to the limited sample size of this group. On the other hand, the results obtained in the present study could possibly indicate that the poorer prognosis of CAP in diabetic patients might be due to other complications associated with diabetes, rather than being directly linked to hyperglycemic conditions. Hence, this discrepancy reveals the requirement for further research in this field, with more pathophysiological details.

In the current study, the length of hospital stay and the frequency of ICU hospitalization were higher in the diabetic patients. In the study of Kornum et al. [20], the mean length of hospital stay in the diabetic patients was not different from the control group. However, Falguera et al. [14], Ibrahem et al. [18], Bhambar et al. [21], and Saibal et al. [22] reported longer hospital stay and increased ICU hospitalization in diabetic patients.

In the current study, the mortality rate in the diabetic patients was not significantly different from that of the control group. Similar to these results herein, in the study of Ibrahem et al. [18], the mortality rate between the groups did not show significant differences. Similar results were reported in a 2020 study by Hespanhol et al. [2] in Portugal, on pneumonia-related deaths and comorbidities, which examined 40,699 patients with pneumonia over the age of 18 as a retrospective cohort. The results of the present study showed that having diabetes does not increase the risk of death from pneumonia; however, in several other studies [14,20,21,26], the mortality rate was significantly higher in the diabetic group.

Finally, in a 2022 metaanalysis of 38 studies, Barmanray et al. [27] found that in adults hospitalized with CAP, hyperglycemia during hospitalization, but not diabetes alone, was linked to an increased risk of in-hospital death and ICU admission. Hyperglycemia was associated with in-hospital mortality (adjusted OR: 1.28, 95% CI: 1.09 to 1.50) and ICU admission (crude OR: 1.82, 95% CI: 1.17 to 2.84). However, there was no association between the diabetes status and in-hospital mortality (adjusted OR: 1.04, 95% CI: 0.72 to 1.51), 30-day mortality (adjusted OR: 1.13, 95% CI: 0.77 to 1.67), or ICU admission (crude OR: 1.91, 95% CI: 0.74 to 4.95). Diabetes was associated with increased mortality in all studies reporting >90-day postdischarge mortality and with a longer length of stay only in studies reporting crude results (OR: 1.50, 95% CI: 1.11 to 2.01). It is important to note that the current study compared only individuals with a confirmed diagnosis of diabetes and those who were completely healthy in terms of diabetes, while excluding individuals with other blood sugar conditions, including prediabetes. As a result, the findings of the current study may differ from the aforementioned metaanalysis in some respects. However, in most cases, a high degree of similarity can be observed between the results.

**Figure 4:**
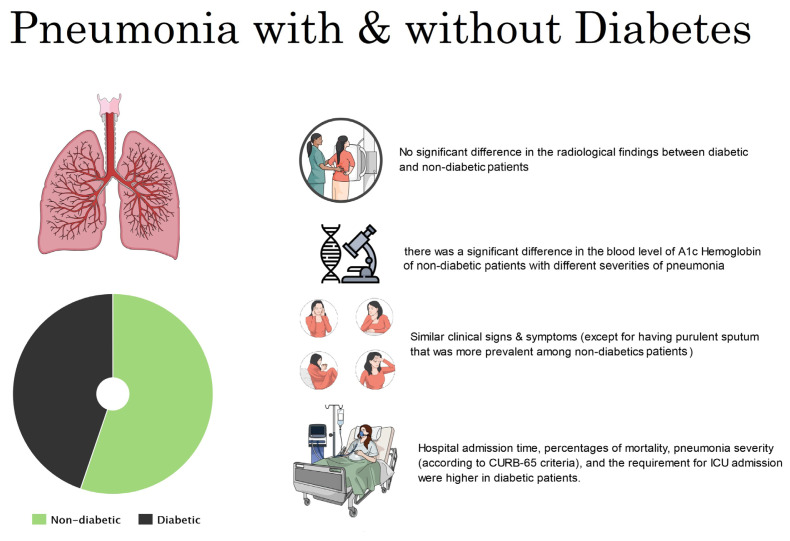
Summary of the important results.

In conclusion, based on the findings herein, diabetic patients with CAP have clinical and radiological findings similar to those of nondiabetic patients, and recovery rates are mostly the same; however, the severity of the disease, average length of hospital stay, and frequency of requiring ICU hospitalization are higher in diabetic patients (Figure 4). In addition, although there was no significant relationship between the severity of pneumonia and the HbA1c level in the diabetic patients, the higher HbA1c level in the nondiabetic patients was significantly correlated with a higher severity of pneumonia.

In analyzing the data, a notably higher prevalence of smoking was observed among the nondiabetic cohort, lending credence to the hypothesis that smoking may serve as a significant predisposing factor for pneumonia in individuals devoid of any underlying or chronic medical conditions. This assertion is further bolstered by the significantly higher presence of purulent sputum in these particular patients. Conversely, the reduced incidence of smokers in the diabetic cohort with pneumonia underscores another crucial finding. The sheer presence of diabetes, independent of other conventional risk factors for pneumonia, appears to substantially heighten the susceptibility to this respiratory ailment. This observation beckons further investigation into the intricate interplay between diabetes and pulmonary health, particularly in the context of infectious diseases.

## Figures and Tables

**Table 1:** Medical histories, clinicoradiological signs, and laboratory characteristics of the patients with pneumonia.

Characteristics	Diabetic patients (n = 77)	Nondiabetic patients (n = 95)	p-value
**Positive smoking history**	10 (13%)	27 (28.4%)	0.014*
**Alcoholism**	1 (1.3%)	0 (0%)	0.448**
**Opium addiction**	3 (3.9%)	2 (2.1%)	0.658**
**HIV+**	1 (1.3%)	3 (3.2%)	0.629**
**Cardiac disease**	18 (23.4%)	11 (11.6%)	0.040*
**Asthma**	4 (5.2%)	2 (2.1%)	0. 410**
**COPD**	11 (14.3%)	13 (13.7%)	0.910*
**Hepatic disease**	0 (0%)	0 (0%)	-
**Malignancies**	0 (0%)	0 (0%)	-
**Immunosuppressive drug consumption**	0 (0%)	0 (0%)	-
**Influenza**	2 (2.6%)	4 (4.2%)	0.639**
**Splenectomy**	0 (0%)	1 (1.1%)	1.00**
**Fever**	41 (53.2%)	53 (55.8%)	0.739*
**Cough**	65 (84.4%)	82 (86.3%)	0.725*
**Purulent sputum**	33 (42.9%)	62 (65.3%)	0.003*
**Bloody sputum**	2 (2.6%)	2 (2.1%)	0.629**
**Shortness of breath**	34 (44.2%)	34 (35.8%)	0.264*
**Chest pain**	0 (0%)	2 (2.1%)	0.503**
**Crackle in the pulmonary auscultation**	44 (57.1%)	46 (48.4%)	0.255*
**Wheezing in the pulmonary auscultation**	3 (3.9%)	4 (4.2%)	1.00**
**Interstitial infiltration (radiologic)**	38 (50.6%)	40 (42.1%)	0.264*
**Lobular infiltration (radiologic)**	21 (27.3%)	32 (33.7%)	0.365*
**Lobar infiltration (radiologic)**	18 (23.4%)	23 (24.2%)	0.898*
**Atelectasis (radiologic**)	1 (1.3%)	0 (0%)	0.448**
**Bronchopneumonia (radiologic)**	0 (0%)	0 (0%)	-
**Pneumatocele (radiologic)**	0 (0%)	0 (0%)	-
**HbA1c (%) (mean ± SD)**	8.86 ± 2.54	5.13 ± 0.33	<0.001***
**FBG (mg/dL) (mean ± SD)**	179.30 ± 58.90	84 ± 9.08	<0.001***
**RBG (mg/dL) (mean ± SD)**	204.82 ± 75.10	105.26 ± 37.48	<0.001***
**ESR (mm/h) (mean ± SD)**	56.45 ± 35.08	47.78 ± 33.35	0.111***
**BUN (mg/dL) (mean ± SD)**	25.65 ± 23.34	23.93 ± 21.16	0.372***
**Creatinine (mg/dL) (mean ± SD)**	1.31 ± 0.85	1.19 ± 0.50	0.698***
**WBC (1000/μL) (mean ± SD)**	10.64 ± 6.31	10.22 ± 5.62	0.939***

* Pearson’s chi-squared test,

** Fisher’s exact test,

*** Mann–Whitney U test.

BUN: blood urea nitrogen, COPD: chronic obstructive pulmonary disease, ESR: erythrocyte sedimentation rate, FBG: fasting blood glucose, HbA1c: A1c hemoglobin, HIV: human immunodeficiency virus, RBG: random blood glucose, WBC: white blood cell.

**Table 2:** Comparison of the sputum smear and culture, blood culture, and blood levels of CRP between the diabetic and nondiabetic patients with pneumonia.

Laboratory variable		Diabetic patients (n = 77)	Nondiabetic patients (n = 95)	p-value
**Sputum (or respiratory secretion suction) smear**	+	0 (0%)	3 (3.2%)	0.254*
	−	77 (100%)	92 (96.8%)	
**Sputum (or respiratory secretion suction) culture**	+	1 (1.3%)	1 (1.1%)	1.00*
	−	76 (98.7%)	94 (98.9%)	
**Blood culture**	+	1 (1.3%)	4 (4.2%)	0.382*
	−	76 (98.7%)	91 (95.8%)	
**CRP**	−	25 (31.6%)	44 (46.3%)	0.060**
	+ −	4 (5.3%)	4 (4.2%)	
	+	26 (34.2%)	26 (27.4%)	
	++	13 (17.1%)	15 (15.8%)	
	+++	9 (11.8%)	6 (6.3%)	

* Pearson’s chi-squared test,

** Mann–Whitney U test

**Table 3:** Frequency distribution of the serum hemoglobin A1C levels in the diabetic and nondiabetic patients with pneumonia and its relation to the severity of pneumonia.

Severity of pneumonia	A1c hemoglobin (%) Mean ± SD	
	Diabetic patients	Nondiabetic patients
**0**	-	4.53 ± 0.44
**1**	6.70 ± 0.11	4.30 ± 0.87
**2**	9.71 ± 2.91	4.95 ± 0.28
**3**	8.53 ± 2.21	5.04 ± 0.43
**4**	9.35 ± 3.07	5.50 ± 0.10
**5**	6.30 ± 2.36	-
**p-value**	0.940*	0.002*

* Kruskal–Wallis one-way analysis of variance

## Data Availability

The data supporting this study’s findings are available from the corresponding author upon reasonable request.
